# Increased uracil misincorporation in lymphocytes from folate-deficient rats

**DOI:** 10.1054/bjoc.2000.1481

**Published:** 2000-11-22

**Authors:** S J Duthie, G Grant, S Narayanan

**Affiliations:** Rowett Research Institute, Greenburn Road, Bucksburn, Aberdeen, AB21 9SB, UK

**Keywords:** folic acid deficiency, methyl-donor deficiency, rat lymphocytes, misincorporated uracil, oxidized DNA bases

## Abstract

The development of certain human cancers has been linked with inadequate intake of folates. The effects of folate deficiency in vivo on DNA stability (strand breakage, misincorporated uracil and oxidative base damage) in lymphocytes isolated from rats fed a diet deficient in folic acid was determined. Because the metabolic pathways of folate and other methyl donors are closely coupled, the effects of methionine and choline deficiency alone or in combination with folate deficiency were determined. Feeding male Hooded Lister rats a folate-free diet for 10 weeks created a moderate folate deficiency (25–50% (approx.) decrease in plasma, red blood cell and hepatic folate concentrations (P < 0.05) and a 20% rise in plasma homocysteine (P < 0.05)). Lymphocyte DNA strand breakage was increased successively in all groups after 4 weeks and 8 weeks on the diet (50–100% (approx.) after 8 weeks). Only low folate specifically and progressively induced uracil misincorporation throughout the study (100% (approx.) after 8 weeks). Neither folate deficiency nor choline/methionine deficiency altered oxidative DNA base damage. In summary, moderate folate deficiency in vivo is associated with a decrease in DNA stability, measured as increased DNA strand breakage and misincorporated uracil. © 2000 Cancer Research Campaign http://www.bjcancer.com


*British Journal of Cancer* (2000) **83**(11), 1532–1537
© 2000 Cancer Research Campaign
doi: 10.1054/ bjoc.2000.1481, available online at http://www.idealibrary.com on IDEAL®

http://www.bjcancer.com

### Increased uracil misincorporation in lymphocytes from folate-deficient rats

**SJ Duthie, G Grant and S Narayanan**

Rowett Research Institute, Greenburn Road, Bucksburn, Aberdeen AB21 9SB, UK

Summary The development of certain human cancers has been linked with inadequate intake of folates. The effects of folate deficiency in vivo on DNA stability (strand breakage, misincorporated uracil and oxidative base damage) in lymphocytes isolated from rats fed a diet deficient in folic acid was determined. Because the metabolic pathways of folate and other methyl donors are closely coupled, the effects of methionine and choline deficiency alone or in combination with folate deficiency were determined. Feeding male Hooded Lister rats a folate-free diet for 10 weeks created a moderate folate deficiency (25–50% (approx.) decrease in plasma, red blood cell and hepatic folate concentrations ($P < 0.05$) and a 20% rise in plasma homocysteine ($P < 0.05$)). Lymphocyte DNA strand breakage was increased successively in all groups after 4 weeks and 8 weeks on the diet (50–100% (approx.) after 8 weeks). Only low folate specifically and progressively induced uracil misincorporation throughout the study (100% (approx.) after 8 weeks). Neither folate deficiency nor choline/methionine deficiency altered oxidative DNA base damage. In summary, moderate folate deficiency in vivo is associated with a decrease in DNA stability, measured as increased DNA strand breakage and misincorporated uracil. © 2000 Cancer Research Campaign http://www.bjcancer.com

**Keywords:** folic acid deficiency; methyl-donor deficiency; rat lymphocytes; misincorporated uracil; oxidized DNA bases

Folate, via its ability to donate or accept one-carbon units, is essential in more than 100 biochemical reactions. As mammals are unable to synthesize folic acid de novo it must be obtained from the diet. Dark green leafy vegetables, fruit juice, liver, bread and cereals are the principal sources of folate in the human diet (Bailey, 1995). However, in Northern Europe, a substantial part of the population does not consume the average recommended folate intake of 200 μg per day (DeBree et al, 1997). Poor folate status has been implicated in the pathology of several human diseases. For example, folate deficiency throughout the periconceptional period of pregnancy is associated with an increased risk of giving birth to a child with neural tube defects (Czeizel, 1993). Similarly, insufficient dietary folate intake is associated with an increased risk of coronary heart disease (Verhoff et al, 1995). Moreover, folate deficiency has been implicated in the development of cancer of the cervix, lung, pancreas and colon (Glynn and Albanes, 1994; Stolzenberg-Solomon et al, 1999).

One proposed mechanism whereby folate deficiency may increase the risk of malignant transformation is by negatively effecting DNA synthesis and repair.

Folic acid is fundamental for the synthesis of purines and the pyrimidine nucleoside thymidine. Deoxyuridine monophosphate (dUMP) is converted to thymidine monophosphate (TMP) by thymidylate synthetase using 5,10-methylenetetrahydrofolate as methyl donor. If folate is limiting, the balance of DNA precursors may be altered leading to dUMP accumulation and incorporation of uracil into DNA in place of thymine. Under normal conditions the DNA repair enzyme, uracil DNA glycosylase, extracts any misincorporated uracil from the DNA strand. Subsequent DNA repair enzymes remove the base-free sugar, causing a transient breakage in the DNA molecule which is sealed by DNA ligase. However, if folate is continually limited, uracil misincorporation and repair may occur continually in a 'catastrophic' repair cycle. This repeated breakage of the DNA molecule might ultimately lead to chromosomal damage and malignant transformation (Reidy, 1987, 1988; Blount and Ames, 1994).

In this study we have investigated the effects of dietary folate deficiency in vivo on DNA stability in rats. As folate is important for the regeneration and metabolism of other cellular methyl sources, these parameters were also measured in rats fed a diet deficient in the methyl donors, methionine and choline.

### MATERIALS AND METHODS

### Materials

LymphoPrep lymphocyte separation medium (LSM) was from Nycomed UK (Birmingham, UK). Ultrapure low melting point (LMP) and electrophoresis grade high melting point (HMP) agarose were from Gibco Life Technologies (Paisley, UK). DAPI (4′,6-diamidine-2-phenylindole dihydrochloride) was obtained from Boehringer Mannheim (Lewes, UK). Uracil DNA glycosylase (1 unit $ml^{-1}$) was from Helena Biosciences (Sunderland, UK). Fetal calf serum (FCS) was from Globepharm Ltd (Surrey, UK). ICN Flow (Irvine, UK) supplied Simultrac Radioassay Kit Vitamin $B_{12}$ [$^{57}$Co] folic acid [$^{125}$I] and Dutch Modified RPMI 1640 medium. All other reagents were from Sigma (Poole, UK).

Endonuclease III and fapyglycosylase were prepared in the laboratory from overproducing plasmid vectors (Asahara et al, 1989).

*Received 27 April 2000*
*Revised 19 July 2000*
*Accepted 28 July 2000*

*Correspondence to:* SJ Duthie

### Animals and diets

Isoenergetic diets containing 112 g casein protein kg$^{-1}$ were formulated (Table 1) as described previously (Grant et al, 1993). Mineral and vitamin mixes were in accordance with NRC recommendations and prepared as before (Grant et al, 1993) with the exception that the vitamin mix was devoid of folic acid and choline chloride. Diets were prepared at 2-week intervals and stored at –20˚C when not in use.

All procedures were carried out in strict accordance with the requirements of UK Animals (Scientific Procedures) Act 1986.

40 male Hooded-Lister (Rowett strain) rats were used. They were weaned at 19 days, group housed and given free access to a lactalbumin-based semi-synthetic control diet (Grant et al, 1993) until they reached 95–100 g (40 days old). They were subsequently individually housed and offered a fixed amount (12 g d$^{-1}$) of the same control diet for 5 days.

Blood samples (tail vein) were collected for 'comet analysis' from 8 untreated rats. These rats were subsequently killed by anaesthetic overdose (halothane) and exsanguination via cardiac puncture, and blood taken for plasma, erythrocyte and lymphocyte preparation. The remaining rats were fed the appropriate experimental diet (Table 1; 8 per diet) exclusively for 10 weeks. They were given a fixed amount of diet daily throughout the study; initially 12 g rat$^{-1}$ d$^{-1}$, increasing to 15 g rat$^{-1}$ d$^{-1}$ after 1 week and to 16.5 g rat$^{-1}$ day$^{-1}$ from 6 weeks onwards. All food was eaten. The amount offered was equivalent to 90–100% of normal free intake of semi-synthetic diet. Water was freely available at all times and the rats were weighed daily. Blood samples (tail vein) were collected from all rats at 4 and 8 weeks. After 10 weeks, the rats were killed by anaesthetic overdose and exsanguination as before and blood collected.

### Isolation of rat lymphocytes for comet analysis

DNA instability was measured in rat lymphocytes at regular intervals throughout the experimental period. Whole blood (approx. 0.5 ml) was collected from the tail vein into Eppendorfs. Blood (30 µl) was resuspended in RPMI 1640 medium supplemented with 10% (v/v) FCS (1 ml), underlain with LymphoPrep (100 µl) and centrifuged at 200 ***g*** for 4.5 min at 4˚C.

### Comet analysis (single cell gel electrophoresis)

DNA instability (strand breaks, misincorporated uracil, oxidized purines and oxidized pyrimidines) was measured using the alkaline comet assay or single cell gel electrophoresis (Duthie et al, 1996; Duthie and Hawdon, 1998). Isolated rat lymphocytes were resuspended in 80 µl of 1% LMP agarose (w/v in PBS, pH 7.4) and pipetted onto a frosted glass microscope slide precoated with 80 µl of 1% HMP agarose. The agarose was allowed to set for 10 min at 4˚C and the slide incubated for 1 h at 4˚C in lysis solution (2.5 M NaCl, 10 mM Tris, 100 mM $Na_2$EDTA and 1% (v/v) Triton X-100 (NaOH to pH 10.0)). The slide was washed 3 times for 5 min each in uracil DNA glycosylase buffer (60 mM Tris-HCl, 1 mM EDTA, 0.1 mg ml$^{-1}$ BSA, pH 8.0) or endonuclease III/fapyglycosylase buffer (40 mM HEPES-KOH, 0.1 M KCl, 0.5 mM EDTA, 0.2 mg ml$^{-1}$ BSA, pH 8.0) and blotted with tissue paper. The gel was covered with either 50 µl appropriate buffer or with uracil DNA glycosylase (misincorporated uracil), or endonuclease III (oxidized pyrimidines) or fapyglycosylase (oxidized purines), sealed with a coverslip and incubated at 37˚C for up to 1 h (see figure legends) in a moist atmosphere. The slides were subsequently aligned in a horizontal gel electrophoresis tank (260 mm wide) containing electrophoresis buffer (0.3 M NaOH and 1 mM $Na_2$EDTA, pH 12.7) for 40 min before electrophoresis at 25 V for 30 min. The slides were washed 3 times for 5 min each at 4˚C in neutralizing buffer (0.4 M Tris-HCl, pH 7.5) and stained with DAPI.

Fluorescently stained nucleoids (5 µg ml$^{-1}$ DAPI stock solution) were scored visually (Duthie and Hawdon, 1998). 100 images per gel (with duplicate gels per slide) were classified according to the intensity of fluorescence in the comet tail and assigned a value of 0, 1, 2, 3 or 4, with 0 representing undamaged cells and 4 representing maximally damaged cells. Accordingly, the total score per gel (in arbitrary units) ranges from 0 to 400. DNA strand breakage is estimated based only on the score obtained from buffer-treated gels. Misincorporated uracil or oxidized pyrimidines and purines are measured by subtracting the visual score obtained from buffer-treated gels from the score obtained after incubation with appropriate enzyme (Duthie et al, 1996; Duthie and Hawdon, 1998). This method of classification has been extensively validated using computerized image analysis (Komet 3.0, Kinetic Imaging Ltd, Liverpool, UK) (Duthie and Hawdon, 1998).

### Preparation of plasma, liver and erythrocytes for measurement of folate, vitamin $B_{12}$ and homocysteine and isolation of lymphocyte 'buffy coat'

At the end of the experimental period the rats were killed as described above, the blood collected into EDTA vacutainers, and spun at 2400 ***g*** for 15 min at 4˚C. The plasma was aliquoted into 1.5 ml plastic tubes, 'snap frozen' in liquid nitrogen and stored at –80˚C for analysis. The lymphocyte-containing 'buffy coat' (approx. 2 ml in the red blood cell layer) was removed into RPMI 1640 medium supplemented with EDTA (4 mM), layered onto an equal volume of LSM and centrifuged at 700 ***g*** for 30 min at room temperature. The lymphocyte layer was decanted and washed twice by centrifuging as described above for 20 min each time. The pellet was resuspended in 90% FCS/10% DMSO (freezing mix) at a final cell density of $3 \times 10^6$ ml$^{-1}$, slowly cooled at –1˚C min$^{-1}$ in polystyrene, and stored at –80˚C. Cell number was determined using a Neubauer Improved Haemocytometer. Erythrocytes reconstituted to initial blood volume with PBS following plasma separation were aliquoted, 'snap frozen' and stored at –80˚C. The liver was removed, 'snap frozen' and stored at –80˚C. When required, the liver was thawed, homogenized on ice in 0.02 M phosphate buffer (4˚C) and diluted in 0.02 M borate buffer (pH 7.5, 4˚C) for immediate folate analysis. Protein was determined using bovine serum albumin as standard (Lowry et al, 1951). Plasma (200 µl), hepatic (100 µl homogenate) and erythrocyte (100 µl) folate and vitamin $B_{12}$ were determined using a commercially available kit [Simultrac Radioassay Kit Vitamin $B_{12}$ ($^{57}$Co) folic acid $^{125}$I) supplied by ICN Flow (Irvine, UK)].

Plasma (200 µl) homocysteine and reduced glutathione (200 µl) were measured by reverse phase HPLC using a DS30 Hcy Homocysteine Assay Kit in combination with a DS30 analyser (Drew Scientific, Barrow-in-Furness, UK).

Samples were reanalysed if the coefficient of variation between duplicates was above 6%.

© 2000 Cancer Research Campaign

### Statistical analysis

Data are presented as mean ± SEM with number of animals in parenthesis. Significant differences between all experimental groups were analysed by ANOVA followed by Tukey's HSD post hoc test using 'Statistical Package for Social Sciences' (SPSS version 8).

### RESULTS

Rats were fed a diet deficient in folic acid (folate-free) and/or in the methyl donors choline (choline-free) and methionine (approx. 70% of requirements) for 10 weeks (Table 1). Folate deficiency had no significant effect on body weight gain (Group B; Table 2). Rats fed either a methionine and choline deficient diet (Group C) or a diet deficient in all three methyl donors (Group D) gained 14% (approx.) less weight than control rats (Table 2). No behavioural or pathological abnormalities were observed in animals that were fed an experimental diet compared with rats fed a control diet.

Several biomarkers of folate status were determined (Table 2). Rats fed a diet deficient only in folic acid for 10 weeks (Group B) had lower plasma folate (approx. 50%), erythrocyte and liver folate (25%) than control rats (Group A). Conversely, rats fed a choline and methionine deficient diet (Group C) had elevated plasma folate levels (approx. 50%). Red blood cell and liver folic acid remained constant in this group. Animals fed a diet deficient in all three methyl donors (Group D) exhibited significantly lower red blood cell and liver folate concentrations than control rats. However, there was no net decrease in plasma folate levels in this group.

Moderate methyl and folate deficiency were associated with significantly increased plasma homocysteine (approx. 20%) in all groups (Table 2).

Plasma $B_{12}$ was elevated in animals fed a choline and methionine deficient diet and in animals deficient in all three nutrients (Table 2). However, folate deficiency itself did not affect plasma $B_{12}$ levels. Hepatic $B_{12}$ concentration did not change in any of the groups throughout the experimental period (Table 2). Similarly, reduced glutathione was unaffected by dietary manipulation (Table 2).

The ability of folate and the methyl donors, choline and methionine to modulate DNA stability was investigated. Lymphocyte DNA strand breakage was induced successively after 4 weeks and 8 weeks on the diets in all deficient groups compared with controls (Figure 1). A higher level of strand breakage was associated with choline and methionine deficiency (Group C) than with folate deficiency alone (Group B) after 4 weeks (Figure 1). The greatest amount of strand breakage was measured in lymphocytes isolated from rats fed the combined folate, choline and methionine deficient diet (Figure 1).

Folate deficiency (Group B) increased uracil misincorporation specifically and progressively over the 8-week trial (Figure 2). Uracil misincorporation was also increased in rats fed the combined deficient diet (Group D), but only after 8 weeks (Figure 2). Choline and methionine deficiency (Group C) did not affect uracil misincorporation at any time throughout the experiment (Figure 2).

**Table 1** Composition of experimental diets (g $kg^{-1}$)

| Diet | A (control) | B (folate-deficient) | C (choline/methionine-deficient) | D (folate/choline/methionine-deficient) |
|---|---|---|---|---|
| Casein | 112 | 112 | 112 | 112 |
| Maize starch | 382 | 382 | 388 | 388 |
| Potato starch | 100 | 100 | 100 | 100 |
| Glucose | 150 | 150 | 150 | 150 |
| Corn oil | 150 | 150 | 150 | 150 |
| Minerals[a] | 50 | 50 | 50 | 50 |
| Vitamins[a] | 50 | 50 | 50 | 50 |
| Sodium silicate | 0.2 | 0.2 | 0.2 | 0.2 |
| L-methionine | 4 | 4 | 0 | 0 |
| Folic acid mix | 0.005 | 0 | 0.005 | 0 |
| Choline chloride | 2 | 2 | 0 | 0 |

[a]Mineral and vitamin mixes were prepared as described (Grant et al, 1993) with the exception that folic acid and choline chloride were absent.

**Table 2** The influence of a methyl-deficient diet on experimental biomarkers

| Group | A (control) | B (folate-deficient) | C (choline/methionine-deficient) | D (folate/choline/methionine-deficient) |
|---|---|---|---|---|
| Body weight gain (g) | 273 ± 13 | 271 ± 10 | 235 ± 5* | 236 ± 7* |
| Plasma folate (ng $ml^{-1}$) | 58.1 ± 4.1 | 30.5 ± 4.2* | 89.5 ± 7.3* | 58.8 ± 5.3 |
| Erythrocyte folate (ng $mg^{-1}$) | 9.2 ± 0.4 | 6.7 ± 0.7* | 8.2 ± 0.4 | 5.9 ± 0.4* |
| Liver folate (ng $mg^{-1}$) | 81.2 ± 6.2 | 60.2 ± 4.7* | 70.9 ± 5.4 | 57.6 ± 4.9* |
| Plasma $B_{12}$ (pg $ml^{-1}$) | 890 ± 38.2 | 970 ± 103 | 1080 ± 55* | 1045 ± 43* |
| Liver $B_{12}$ (pg $ml^{-1}$) | 270 ± 14 | 294 ± 32 | 290 ± 23 | 278 ± 20 |
| Plasma homocysteine (µM) | 3.20 ± 0.1 | 3.85 ± 0.1* | 3.93 ± 0.3* | 4.05 ± 0.3* |
| Plasma GSH (µM) | 19.1 ± 0.8 | 19.6 ± 0.8 | 20.7 ± 0.6 | 19.8 ± 0.8 |
| Lymphocytes ($10^4$ $ml^{-1}$) | 288 ± 14.3 | 243 ± 9.6* | 225 ± 9.6* | 193 ± 5.6* [+ #] |

Analyses were performed at the end of the experiment (week 10). Results are mean ± SEM for $n$ = 8. * $P$ < 0.05, where significance refers to differences between Group A and Groups B–D. + $P$ < 0.0005, where significance refers to differences between Group B and Group D. # $P$ < 0.01, where significance refers to differences between Group C and Group D.

 *© 2000 Cancer Research Campaign*

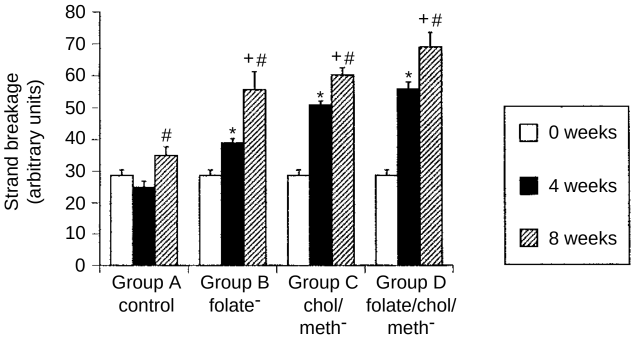

**Figure 1** The effect of methyl donor deficiency on DNA strand breakage in rat lymphocytes. Rats were either fed a control diet (Group A) or a diet deficient in folic acid (Group B), choline and methionine (Group C) or a diet deficient in all 3 methyl donors (Group D) for up to 10 weeks. DNA strand breakage was measured in isolated rat lymphocytes. Two rats from each of the four experimental treatment groups were analysed together on four separate occasions per timepoint. Results are mean ± SEM ($n$ = 8). *$P$ < 0.00002, where significance refers to differences between Group A and Groups B–D after 4 weeks on the diet. + $P$ < 0.005, where significance refers to differences between Group A and Groups B–D after 8 weeks on the diet. # $P$ < 0.02, where significance refers to differences for each group between strand breakage after 4 weeks and 8 weeks on the same diet

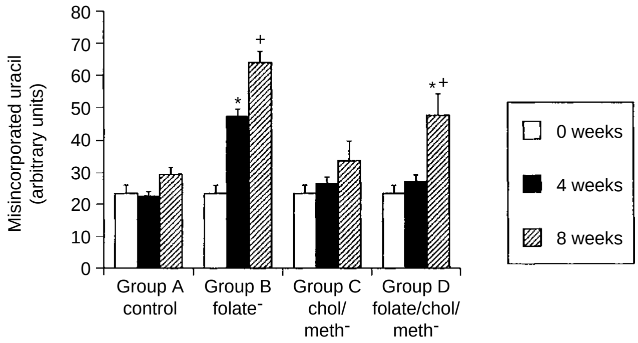

**Figure 2** The effect of methyl donor deficiency on uracil misincorporation in rat lymphocytes. Rats were either fed a control diet (Group A) or a diet deficient in folic acid (Group B), choline and methionine (Group C) or a diet deficient in all 3 methyl donors (Group D) for up to 10 weeks. Uracil was measured in isolated rat lymphocytes. Two rats from each of the four experimental treatment groups were analysed together on four separate occasions per timepoint. Results are mean ± SEM ($n$ = 8). *$P$ < 0.0002, where significance refers to differences in uracil levels between rats fed a control diet (Group A) for 4 weeks and rats fed either a diet deficient in folic acid (Group B) or a diet deficient in folic acid, choline and methionine (Group D). + $P$ < 0.01, where significance refers to differences in uracil levels between rats fed a diet deficient in folic acid (Group B) for 4 weeks or 8 weeks or a diet deficient in folic acid, choline and methionine for 4 weeks or 8 weeks (Group D)

Neither folate deficiency nor choline/methionine deficiency altered the extent of oxidative DNA base damage measured in rat isolated lymphocytes at the end of the 10-week experiment (Figure 3). However, while there was a significant decrease in the level of oxidized purines at this time in lymphocytes from animals fed the combined deficient diet (Group D) (Figure 3), the level of oxidized pyrimidines from this group remained unchanged (Figure 3).

All of the diets caused a fall in the numbers of lymphocytes isolated from whole rat blood; this was particularly evident in rats fed the combined folate, choline and methionine deficient diet (Group D) (Table 2). Viability (typically >85% as measured by Trypan blue exclusion) was similar in all groups (data not shown).

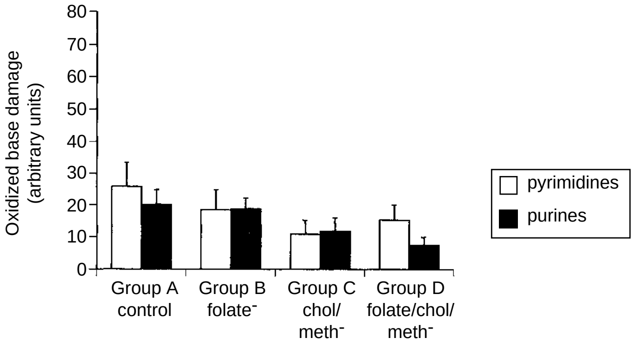

**Figure 3** The effect of methyl donor deficiency on oxidized DNA bases in rat lymphocytes. Rats were either fed a control diet (Group A) or a diet deficient in folic acid (Group B), choline and methionine (Group C) or a diet deficient in all 3 methyl donors (Group D) for up to 10 weeks. Oxidized pyrimidines and oxidized purines were measured in isolated rat lymphocytes. Two rats from each of the four experimental treatment groups were analysed together on four separate occasions per timepoint. Results are mean ± SEM ($n$ = 8). *$P$ < 0.03, where significance refers to differences in oxidized purine levels between rats fed a control diet (Group A) for 8 weeks and rats fed a diet deficient in folic acid, choline and methionine (Group D)

### DISCUSSION

Low intake of the B vitamin folic acid has been implicated in the development of certain epithelial cell cancers (Glynn and Albanes, 1994; Stolzenberg-Solomon et al, 1999). Folate deficiency has been hypothesized to increase cancer risk by perturbing DNA synthesis and repair and negatively affecting DNA stability (Blount et al, 1997; Duthie, 1999; Kim, 1999).

Folate is important in the metabolism and regeneration of the dietary methyl donors choline and methionine. In this study we have investigated the effects of in vivo folate, choline and methionine deficiency on DNA stability in rat lymphocytes. Lymphocytes are exquisitely sensitive to folate depletion and have proved to be a useful surrogate biomarker for investigating the influence of nutrition in human population biomonitoring (Duthie et al, 1996).

DNA strand breakage increased progressively in rats fed either a folate- or a methionine- and choline-deficient diet. Moreover, depleting the diet of methionine and choline in addition to folate had an even greater effect on strand breakage, suggesting that DNA strand breakage, as a result of methyl donor deficiency, occurs relatively non-specifically. Folate depletion in vitro causes strand break accumulation in repair-deficient Chinese hamster ovary cells (Melnyk et al, 1999), while methyl deficiency in vivo increases DNA strand breakage in rat liver (Pogribny et al, 1995). Methyl donor-deficiency-induced disruption in DNA repair mechanisms could explain the general increase in DNA strand breakage.

In contrast, increased uracil misincorporation (above endogenous levels) was detected only in lymphocytes from rats consuming a low folate diet. Folate deficiency inhibits thymidine synthesis so that the ratio of intracellular dUTP:dTTP is increased and uracil is misincorporated into DNA (James et al, 1997). 'Catastrophic repair' of uracil destabilizes the DNA molecule, leading to chromosomal aberrations and malignant transformation (Reidy, 1987, 1988; Melnyk et al, 1999). Myeloid cells made folate-deficient in vitro and bone marrow cells isolated from megaloblastic anaemia patients contain high levels of uracil (Wickramasinghe and Fida, 1993, 1994). Similarly, the chemotherapeutic agent methotrexate (a dihydrofolate reductase inhibitor) significantly increases the intracellular ratio of dUTP to dTTP in human lymphoid cells and induces uracil misincorporation into DNA in vitro (Goulian et al, 1980). Uracil is also increased in liver DNA from hepatectomized rats exposed to methotrexate or untreated rats fed a combined folate/methyl-deficient diet (Blount and Ames, 1994; Pogribny et al, 1997).

In the present study, choline and methionine deficiency alone did not increase uracil levels in lymphocyte DNA suggesting that this mechanism is specific for folate deficiency.

While folate deficiency alone induced a progressive and time-dependent increase in uracil in lymphocyte DNA, surprisingly, rats consuming the combined folate/methyl-low diet displayed elevated uracil levels only after 8 weeks on the diet. Uracil misincorporation in these groups is probably linked with folate concentration. Plasma, red blood cell and liver folate concentrations were all significantly reduced in rats fed only a folate-free diet; the animals, which showed a dramatic increase in uracil levels after just 4 weeks. In rats fed the combined deficient diet, erythrocyte and hepatic folate stores were similarly affected but plasma folate remained unchanged (Table 2). In this group, the induction of uracil was delayed (8 weeks) compared with rats fed only the folate-free diet. Moreover, choline and methionine deficiency alone did not affect folate stores and indeed significantly increased plasma folate. Uracil misincorporation was not increased in this group. These results also support the observation that DNA strand breakage is not entirely dependent on folate concentration but reflects methyl-donor status in general. Alternatively, it may be that the combined folate, methionine and choline deficiency was intensely stressful on the lymphocytes, inducing apoptosis and effectively removing severely damaged cells from the sample population. Indeed, the level of circulating lymphocytes recovered was significantly reduced in rats made folate (15%) or choline and methionine deficient (22%) in the present study. Furthermore, lymphocyte number was decreased more than 30% in rats fed the diet deficient in folate, methionine and choline. Severe folate deficiency in mice induces p53 expression and apoptosis in splenic erythroblasts (Koury and Horne, 1994). Moreover, uracil and p53 protein accumulates in these cells (Koury et al, 1995). Similarly, hepatocytes from folate/methionine/choline-deficient rats or immortalized rat hepatocytes grown in choline-deficient media undergo apoptosis (Albright et al, 1997; James et al, 1997). Hepatocytes, selected to survive under choline-deficient conditions, are capable of malignant transformation in vivo (Albright et al, 1997).

Choline deficiency may induce hepatocarcinogenesis by inhibiting phase II detoxifying enzyme activities and increasing oxygen-radical-mediated membrane peroxidation and DNA damage (Nakae et al, 1998). However, in our study, oxidative DNA base damage (pyrimidines and purines) was unaffected by folate or choline and methionine deficiency and, likewise, declined in animals fed the combined deficient diet. Again, this may be due to apoptosis of severely damaged lymphocytes in this group. Moreover, reduced glutathione concentrations were unaltered in all the experimental groups compared with controls. These data suggest that folate and methyl-donor deficiency, at this level, do not induce oxidative stress. Moreover, they support the theory that folate deficiency induces uracil misincorporation specifically.

In summary, consumption by rats of a folate-free diet for 10 weeks created a moderate folate deficiency, as indicated by a 25–50% decrease in plasma, red blood cell or hepatic folate concentrations and a 20% rise in plasma homocysteine, a functional marker for folate status. DNA stability was modulated by folic acid. Folate deficiency markedly increased DNA strand breakage and misincorporated uracil in isolated rat lymphocytes. Dietary methyl donor-deficiency increased DNA strand breakage but did not elevate uracil levels in DNA, indicating the specificity of this lesion in folate deficiency.

### ACKNOWLEDGEMENTS

The World Cancer Research Fund and The Scottish Executive Rural Affairs Department (SERAD) supported this work. The authors would like to thank Mr N Vaughan for production of DNA repair enzymes.

### REFERENCES

Albright CD, Liu R, Mar MH, Shin OH, Vrablic AS, Salganik RI and Zeisel SH (1997) Diet, apoptosis and carcinogenesis. *Adv Exp Med Biol* **422**: 97–107

Asahara H, Wistort PM, Bank JF, Bakerian RH and Cunningham RP (1989) Purification and characterisation of *Escherichia coli* endonuclease III from the cloned *n*th gene. *Biochemistry* **28**: 4444–4449

Bailey LB (1995) Folate requirements and dietary recommendations. In: Bailey LB (ed) *Folate in Health and Disease*. Marcel Dekker, Inc, New York, pp. 123–151

Blount BC and Ames BN (1994) Analysis of uracil in DNA by gas chromatography–mass spectrometry. *Anal Biochem* **219**: 195–200

Blount BC, Mack MM, Wehr CM, MacGregor JT, Hiatt RA, Wang G, Wickramasinghe SN, Everson RB and Ames BN (1997) Folate deficiency causes uracil misincorporation into human DNA and chromosomal breakage: implications for cancer and neuronal damage. *Proc Natl Acad Sci USA* **94**: 3290–3295

Czeizel AE (1993) Prevention of congenital abnormalities by periconceptional multivitamin supplementation. *BMJ* **306**: 1645–1649

DeBree A, van Dusseldorp M, Brouwer IA, van het Hof KH and Steegers-Theunissen RPM (1997) Review: Folate intake in Europe: recommended, actual and desired intake. *Eur J Clin Nutr* **51**: 643–660

Duthie SJ (1999) Folic acid deficiency and cancer: mechanisms of DNA instability. *Brit Med Bull* **55**: 578–592

Duthie SJ and Hawdon A (1998) DNA stability (strand breakage, uracil misincorporation, and defective repair) is increased by folic acid depletion in human lymphocytes *in vitro*. *FASEB J* **12**: 1491–1497

Duthie SJ, Ma A, Ross MA and Collins AR (1996) Antioxidant supplementation decreases oxidative DNA damage in human lymphocytes. *Cancer Res* **56**: 1291–1295

Glynn SA and Albanes D (1994) Folate and cancer: a review of the literature. *Nutr Cancer* **22**: 101–119

Goulian M, Bleile B and Tseng BY (1980) The effect of methotrexate on levels of dUTP in animal cells. *J Biol Chem* **255**: 10630–10637

Grant G, Dorward PM and Pusztai A (1993) Pancreatic enlargement is evident in rats fed diets containing raw soyabean (*Glycine max*) or cowpea (*Vigna unguiculata*) for 800 days but not in those given diets based on kidney bean (*Phaseolus vulgaris*) or lupinseed (*Lupinus angustifolius*). *J Nutr* **123**: 2207–2215

James SJ, Miller BJ, Basknakian AG, Pogribny IP, Pogribny M and Muskhelishvili L (1997) Apoptosis and proliferation under conditions of deoxynucleotide pool imbalance in liver of folate/methyl deficient rats. *Carcinogenesis* **18**: 287–293

Kim Y-I (1999) Folate and carcinogenesis: evidence, mechanisms and implications. *J Nutr Biochem* **10**: 6–88

Koury MJ and Horne DW (1994) Apoptosis mediates and thymidine prevents erythroblast destruction in folate deficiency anaemia. *P.N.A.S.* **91**: 4067–4071

Koury MJ, Horne DW, Blount BC and Ames BN (1995) Erythroblasts undergoing apoptosis due to folate deficiency misincorporate uracil into DNA and accumulate the tumour suppressor protein p53. *Blood* **86**: 2324

Lowry OH, Rosenburgh NJ, Farr AL and Randall RJ (1951) Protein measurement with folin phenol reagent. *J Biol Chem* **193**: 265–270

Melnyk S, Pogribny M, Miller BJ, Basnakian AG, Pogribny IP and James SJ (1999) Uracil misincorporation, DNA strand breaks and gene amplification are associated with tumorigenic cell transformation in folate deficient/repleted Chinese hamster ovary cells. *Cancer Letts* **146**: 35–44

*© 2000 Cancer Research Campaign*

Nakae D, Kotake Y, Kishida H, Hensley KL, Denda A, Kobayashi Y, Kitayama W, Tsujiuchi T, Sang H, Stewart CA, Tabatbaie T, Floyd RA and Konoshi Y (1998) Inhibition by phenyl N-tert-butyl nitrone of early phase carcinogenesis in the livers of rats fed a choline-deficient, L-amino acid-defined diet. *Cancer Res* **58**: 4548–4551

Pogribny IP, Basnakian AG, Miller BJ, Lopatina NG, Poirier LA and James SJ (1995) Breaks in genomic DNA and within the p53 gene are associated with hypomethylation in livers of folate/methyl deficient rats. *Cancer Res* **55**: 1894–1901

Pogribny IP, Muskheloshvili L, Miller BJ and James SJ (1997) Presence and consequence of uracil in preneoplastic DNA from folate/methyl-deficient rats. *Carcinogenesis* **18**: 2071–2076

Reidy JA (1987) Folate- and deoxyuridine-sensitive chromatid breakage may result from DNA repair during G2. *Mutat Res* **192**: 217–219

Reidy JA (1988) Role of deoxyuridine incorporation and DNA repair in the expression of human chromosomal fragile sites. *Mutat Res* **200**: 215–220

Stolzenberg-Solomon RZ, Albanes D, Nieto FJ, Hartman TJ, Tangrea JA, Rautalahi M, Selhub J, Virtamo J and Taylor PR (1999) Pancreatic cancer risk and nutrition-related methyl group availability indicators in male smokers. *J Natl Cancer Inst* **91**: 535–541

Verhoff P, Stampfer MJ, Buring JE, Gaziano JM, Allen RH, Stabler SP, Reynolds RD, Kok FJ, Hennekens CH and Willet W (1995) Homocysteine metabolism and risk of myocardial infarction: relation with vitamins B6, $B_{12}$ and folate. *Am J Epidemiol* **143**: 845–859

Wickramasinghe SN and Fida S (1993) Misincorporation of uracil into the DNA of folate- and $B_{12}$-deficient HL60 cells. *Eur J Haematol* **50**: 127–132

Wickramasinghe SN and Fida S (1994) Bone marrow cells from vitamin $B_{12}$- and folate-deficient patients misincorporate uracil into DNA. *Blood* **83**: 1656–1661

*© 2000 Cancer Research Campaign*
